# Polymorphisms in XPD and ERCC1 Associated with Colorectal Cancer Outcome

**DOI:** 10.3390/ijms14024121

**Published:** 2013-02-19

**Authors:** Ming-Yii Huang, Jaw-Yuan Wang, Meng-Lin Huang, Hui-Jen Chang, Shiu-Ru Lin

**Affiliations:** 1 Department of Radiation Oncology, Cancer Center, Kaohsiung Medical University Hospital, Kaohsiung 807, Taiwan; E-Mail: miyihu@gmail.com; 2 Department of Radiation Oncology, Faculty of Medicine, College of Medicine, Kaohsiung Medical University, Kaohsiung 807, Taiwan; 3 Division of Gastrointestinal and General Surgery, Department of Surgery, Cancer Center, Kaohsiung Medical University Hospital, Kaohsiung 807, Taiwan; E-Mail: cy614112@ms14.hinet.net; 4 Department of Surgery, Faculty of Medicine, Graduate Institute of Medicine, Kaohsiung 807, Taiwan; 5 Department of Medical Genetics, College of Medicine, Kaohsiung Medical University, Kaohsiung 807, Taiwan; 6 Graduate Institute of Clinical Medicine, College of Medicine, Kaohsiung Medical University, Kaohsiung 807, Taiwan; 7 Division of Colorectal Surgery, Department of Surgery, ZuoYing Armed Forces General Hospital, Kaohsiung 813, Taiwan; E-Mail: kmu7158@gmail.com; 8 School of Medical and Health Science, Fooyin University, Kaohsiung Hsien 831, Taiwan; E-Mail: hu840112@gmail.com; 9 Department of Medical Research, Fooyin University Hospital, Pingtung County 928, Taiwan

**Keywords:** genetic polymorphism, XPD, ERCC1, colorectal cancer, regional recurrence

## Abstract

Using the comprehensive approach to selecting polymorphisms to date, we sought to examine whether recurrence in colorectal cancer was associated with inherited variation in three genes involved in DNA repair and cell proliferation. Three polymorphisms, which are excision repair cross-complementation 1 (ERCC1), xeroderma pigmentosum group D (XPD) and epidermal growth factor receptor (EGFR), were assessed in 257 postoperative stage II/III CRC patients with 5-fluorouracial chemotherapy in Taiwan. In addition, the correlations between genetic polymorphisms and patients’ clinicopathological features were investigated. Genotypes of XPD codon751 A/A and ERCC1 codon118 T/T were associated with regional recurrence in a statistically significant way (*p* = 0.018). Patients who carried XPD AA and ERCC1 TT genotypes demonstrated a significantly greater regional recurrence risk (OR = 5.625, 95% CI, 1.557–20.32). Inherited variation in XPD and ERCC1 was associated with outcome in patients with colorectal cancer in Taiwan. As the significant association of single-nucleotide polymorphisms has not been studied previously in colorectal cancer, these findings suggest novel sites of variation, in part explaining the range of treatment responses seen in this disease.

## 1. Introduction

Colorectal cancer (CRC) remains one of the leading causes of cancer mortality worldwide. Each year, nearly 1,000,000 new cases of CRC are diagnosed and there are 500,000 deaths from CRC [1]. The primary treatment for colorectal cancer (CRC) is resection of the primary tumor. After surgery, patients are frequently administered adjuvant chemotherapy to eliminate cancer cells that may have metastasized [2]. Despite undergoing chemotherapy, CRC remains the third major cause of cancer-related death in Taiwan, accounting for >3000 deaths per year [3]. The overall five-year survival is 50%–60% in European countries [4], whose result is similar to that in Taiwan [5]. The primary cause of death is distant and loco-regional relapses. To date, most studies regarding prognosis of CRC have focused on tumor characteristics, but less on patients’ characteristics.

Genetic polymorphisms in DNA-repairing enzymes [6,7] and carcinogenesis [8–11] have been linked to inter-individual differences in the tumor phenotype aggressiveness, therapeutic response and disease prognosis. Several studies have investigated various genetic polymorphisms related to prognosis of cancers. For instance, in Caucasian patients with advanced CRC who are treated with oxaliplatin, 5FU and leucovorin (LV), excision repair cross-complementation 1 (ERCC1) codon118 T/T, X-ray cross-complementing1 (XRCC1) (Arg→Gln substitution in exon 10), excision repair cross-complementation 2 (ERCC2) codon 751 A/C and ERCC2 codon 751 C/C genotypes are independently associated with poor progression-free survival and short-term survival [12,13]. Epidermal growth factor receptor (EGFR) plays a central role in a wide variety of cellular functions, including cell proliferation, migration, adhesion, differentiation and survival; therefore, upregulation of EGFR has been found in several epithelial tumors, including CRC [14]. In Caucasian patients with locally advanced rectal cancer, the higher risk of local recurrence was seen in patients who possessed a EGFR polymorphism (codon 497 Arg(G) allele) [15]. Polymorphisms of EGFR gene had been analyzed in genomic DNA from 318 metastatic colon cancer patients; females with a polymorphism located at the codon 497 (HER-1 R497K) Arg/Arg(G/G) variant of the gene had better overall survival when compared with the Lys/Lys (A/A) and/or Arg/Lys (G/A) variants [11]. The possible explanations of these contradictory findings of two previous studies are the gender-related survival differences associated with EGFR polymorphisms.

Conventional regimens for treating cancer patients with chemotherapy or radiotherapy do not account for interpatient variability in the polymorphisms of particular target genes. Such variability results in unpredictable tumor responses and host toxicity. In the present study, three polymorphisms located in DNA-repair genes (ERCC1 and XPD) and cell proliferation and differentiation related gene (EGFR) were assessed in 257 Taiwanese CRC patients who underwent potentially radical curative surgery using a polymerase chain reaction-restriction fragment-length polymorphism (PCR-RFLP) technique and DNA sequencing. In addition, the correlations between genetic polymorphisms and patients’ clinicopathological features were investigated to elucidate the prevalence of genetic polymorphisms and the feasibility of developing a gene predictor of clinical outcome for Taiwanese colorectal patients. The ability to predict which patients are likely to have a poor prognosis will significantly influence the design of effective treatment regimens.

## 2. Results and Discussion

### 2.1. Results

#### 2.1.1. Patient Characteristics

Of 257 CRC patients, 139 were male (54.1%) and 118 were female (45.9%). One hundred and nineteen patients (46.3%) were aged <60 years, and 138 (53.7%) were ≥60 years (range, 36–71 years; median age, 61.5 ± 11.3 years). The primary tumor location of 152 cases (59.1%) was the colon and of 105 (40.9%), the rectum. The majority of patients had T3 to T4 tumor invasion (80.9%), lymph node metastasis (68.9%) and well and moderately differentiated histological findings (80.5%). Twenty-four cases (9.3%) developed regional recurrence during follow-up. Table 1 lists the clinicopathological characteristics of patients and tumors.

#### 2.1.2. Genotype Frequency

The genotype frequencies of the three genetic polymorphisms in this study were in Hardy-Weinberg equilibrium, and their distributions are shown in Table 2. For ERCC1 codon118, 142 (55.3%) were C/C, 100 (38.9%) were C/T and 15 (5.8%) were T/T genotype carriers. The frequency of the C allele and T allele were 384 (74.7%) and 130 (25.3%). For XPD codon751, 190 (73.9%) were A/A and 66 (25.7%) were A/C genotype carriers and one (0.4%) was a C/C genotype carrier. For EGFR codon497, 52 (20.2%) were G/G, 120 (46.7%) were G/A and 85 (33.1%) were A/A genotype carriers.

#### 2.1.3. Correlation between Genetic Polymorphisms and Clinicopathological Data

This study assessed correlations between the genetic polymorphisms of three genes (including ERCC1, XPD and EGFR) and clinicopathological features of 257 CRC patients (Table 3). No statistically significant correlations existed between genotype distributions and sex, tumor location, depth of tumor invasion, lymph node metastasis, cancer stage or histology (all *p* > 0.05).

#### 2.1.4. Correlation between Regional Recurrence and Clinicopathological Data

No statistical correlations existed between regional recurrence and age, sex, tumor size, depth of tumor invasion, lymph node metastasis, differentiation, tumor location and vascular and perineural invasion (*p* > 0.05; Table 4). CRC patients with regional recurrence have a risk of death 3.068-times greater than those patients without regional recurrence (*p* = 0.009; 95% CI, 1.279–7.362).

#### 2.1.5. Correlation between Regional Recurrence and Gene Expression

The correlations between genetic polymorphisms and patients with or without regional recurrence were examined. CRC patients with ERCC1 codon118 T/T and XPD codon751 A/A genotypes have a risk of regional recurrence 5.625-times greater than those patients without these two genotypes (*p* = 0.018; 95% CI, 1.557–20.32), as shown in Table 5.

### 2.2. Discussion

Despite aggressive surgical resections and a combination of chemotherapy or radiotherapy, the prognosis for relapse in CRC patients is still dismal [5,16,17]. Disease-free interval and progression-free survival could be taken as the best predictors of long-term cure and prognosis in CRC [18]. To date, most studies regarding prognosis of postoperative CRC regional recurrence have focused on tumor characteristics, but less on host-related characteristics [16,17,19–21]. The factors increasing local recurrence rates of CRC should be clearly described. The highest quintile of Western diet (compared to the lowest quintile) can increase the hazard ratio of local/regional recurrence by about 2.5-fold [22]. Exercise can decrease the hazard ratio for colon cancer-specific mortality to about 0.50 [23,24]. On the other hand, in a study of 44,788 pairs of twins, statistically significant effects of heritable factors (polymorphisms, *etc.*) were found to account for 35% of the cases of colon cancer, so that polymorphisms are clearly important in colon cancer [25]. Mention should also be made of the particular importance of decreased expression of ERCC1 in progression to colon cancer, shown in recent studies. A 2012 report indicates that epigenetic repression of ERCC1 is found in 40% of about 1 million crypts in large field defects surrounding colon cancers (in which progression to cancer occurred) and in 100% of colon cancers themselves [26]. Local and systemic treatment modalities, like preoperative chemoradiotherapy, should be planned for patients carrying these risk factors.

Some host-related factors may predict the risk of metastasis after surgery of CRC [27]. There is accumulated evidence suggesting that the genetic polymorphisms involved in metabolizing enzymes, DNA repair, cell growth, differentiation and carcinogenesis have been linked to inter-individual differences of therapeutic effect in CRC [28]. This genetic polymorphism detection may permit better selection of patients suitable for adjuvant therapy [29]. Genetic analyses appear to be a promising tool in the development of personalized treatment plans for CRC [8,30]. Nevertheless, a comprehensive analysis of genetic polymorphisms in Taiwanese CRC patients with poor prognostic factors remains rare. This study aimed to examine the feasibility of developing a multigene predictor of poor prognosis in Taiwanese CRC patients, and it will be helpful in identifying those who may need aggressive treatment.

In the current study, we have attempted to move beyond single gene polymorphisms to a more comprehensive evaluation to identify genomic variants and patterns that may help predict CRC prognosis. The *in vitro* studies suggest a C to T transition at codon 118 of the ERCC1 gene associated with higher ERCC1 mRNA levels resulting in resistance to platinum drugs [31]. However, the clinical data regarding the assumed relationship between ERCC1 codon 118 polymorphism and platinum sensitivity is controversial [13,32,33]. Metastatic CRC patients with ERCC1 codon 118 C/T or T/T genotypes tended to have a marked increase of ERCC1 protein expression levels and had shorter progression-free survival and overall survival than patients with the other polymorphisms [7]. Codon 118 C→T polymorphism of ERCC1 was identified as an independent prognostic factor in Asian CRC patients [7]. In Caucasian advanced CRC patients who are treated with oxaliplatin, 5FU and leucovorin (LV), ERCC1 118T/T, XPD 751A/C and XPD 751C/C genotypes are independently associated with poor progression-free survival and short-term survival [12,13]. In the results of the present study, we also found that ERCC1 codon 118T/T combined with XPD codon 751A/A was associated with increased recurrence risk in postoperative Taiwanese CRC patients. The possible mechanism of the combination of ERCC1 codon 118T/T and XPD codon 751A/A and the increased recurrence risk relate to poor chemotherapy efficacy in combination with DNA repair system impairment, which led to cancer recurrence.

## 3. Experimental Section

### 3.1. Clinical Samples Collection

Enrolled in this study were originally 257 American Joint Commission on Cancer/International Union Against Cancer (AJCC/UICC) stage II–III CRC patients (median age, 61.5 ± 11.3 years) who were admitted to the Department of Surgery at Fooyin University Hospital, ZuoYing Armed Forces General Hospital and Kaohsiung Medical University Hospital for elective surgery from January 2006 to June 2009. Patients with other malignant diseases noted in the medical history were excluded. Patients, who were required to be at least 18 years of age with a life expectancy of 3 months, have an Eastern Cooperative Oncology Group (ECOG) performance status of 0 to 2. Written informed consent was obtained from all subjects and/or guardians for use of their blood samples. This study was approved by the Institutional Review Board of Kaohsiung Medical University Hospital and was not supported by any commercial company. To avoid contamination of skin cells, sampled blood was taken via an intravenous catheter, and the first few milliliters of blood were discarded. Total RNA was immediately extracted from the peripheral whole blood, and then it served as a template for complementary DNA (cDNA) synthesis. The institutional review boards of the three hospitals approved the acquisition of samples, as well as their subsequent use. Written informed consent was obtained from all participants.

Pretreatment evaluation included a complete medical and clinical physical examination, baseline measurement of tumor size based on computed tomography scans, X-ray or other radiographic means and serum biochemistries. Patients were administered six 8-week cycles of adjuvant chemotherapy. Each cycle consisted of leucovorin 500 mg/m^2^ administered as a 2-h infusion and given weekly for six doses and 5FU 500 mg/m^2^ administered as an intravenous bolus 1 h after the start of leucovorin infusion and repeated weekly for 6 doses. This cycle was then repeated after a 2-week rest period. Postoperative surveillance consisted of medical history, physical examination and laboratory studies at 3-month intervals. Abdominal ultrasonography or computed tomography was performed at 6-month intervals, and chest radiography, bone scans and total colonoscopy were performed annually. Patients were followed up at 3-month intervals for 2 years and at 6-month intervals thereafter.

The follow-up endpoint was December 2010. The median follow-up time was 36.4 months (range, 24–46 months). This follow-up time is long enough to draw conclusions regarding regional recurrence [34]. Clinical stage and pathological features of primary tumors were defined according to the criteria of the American Joint Commission on Cancer/International Union Against Cancer (AJCC/UICC) [35]. Development of new post-operative tumor growth restricted to the anastomosis or the region of the primary operation was defined as postoperative regional recurrence.

### 3.2. Polymorphisms Selection, DNA Extraction and Genotyping

In our previous study results, we found that XPD, ERCC1 and EGFR gene polymorphisms were related to stage II–III and metastatic CRC prognosis [36–38]. Constitutional gene polymorphisms were analyzed via DNA extraction from 4 mL peripheral blood using a PUREGENE^®^ DNA Isolation Kit (Gentra Systems, Inc., Minneapolis, MN, USA). All genomic DNA from patients were examined using the polymerase chain reaction-restriction fragment length polymorphism (PCR-RFLP) approach to determine the genotypes of ERCC1, XPD and EGFR. Following digestion with suitable restriction enzymes, PCR fragments were separated on a 2.5%–3.0% agarose gel and visualized after ethidium bromide staining. This study also utilized the automated sequencing approach to confirm PCR-RFLP results. All primers utilized in this study were designed by using Primer 3 freeware [39]. Table 6 presents the primer sequences and restriction enzymes.

### 3.3. Statistical Analysis

The observed numbers of each genotype were compared with those expected for Hardy-Weinberg equilibrium (HWE) using the χ^2^ test. All data were analyzed using the Statistical Package for the Social Sciences Version 14.0 (SPSS, Inc., Chicago, IL, USA). The correlation between each polymorphism and survival was assessed using the hazard risk ratio and a 95% confidence interval (CI). To clarify the clinical significance of these combined genotypes as predictors of prognosis, a multivariate adjustment was performed by multivariate Cox proportional hazards regression analysis. Overall, statistical significance was defined as *p*-value < 0.05. In the tests with multiple comparisons, the Bonferroni correction was applied to the significance level in which a type I error of 0.0167 (0.05/3) was used.

## 4. Conclusions

In conclusion, when three unfavorable genotypes of ERCC1 TT and XPD AA are present, it seems possible to identify CRC patients who suffer the risk of regional recurrence. Identifying risk genes can be useful in predicting the genetic risk of CRC recurrence. Selecting an optimal therapeutic modality on the basis of individual genetic information may present an innovative strategy. Future prospective studies incorporating larger numbers of patients will enable us to determine the facilitation of ERCC1 TT and XPD AA in choosing a tailored therapeutic strategy for poor prognostic CRC patients.

## Figures and Tables

**Table 1:** Clinicopathological features for 257 colorectal cancer patients.

Characteristics	Total cases No.	%
Gender		

Male	139	54.1%
Female	118	45.9%
Age (years)		

<60	119	46.3%
≥60	138	53.7%
Tumor Size		

<5cm	156	60.7%
≥5cm	101	39.3%
Location		

Colon	152	59.1%
Rectum	105	40.9%
Invasive extent		

T1–T2	49	19.1%
T3–T4	208	80.9%
Lymph node status		

N0	80	31.1%
N1–N3	177	68.9%
Stage (UICC) a		

II	60	23.3%
III	197	76.6%
Histologic differentiation		

Well/moderate	207	80.5%
Poorly	50	19.5%
Vascular invasion		

Negative	148	57.6%
Positive	109	42.4%
Perineural invasion		

Negative	204	79.4%
Positive	53	20.6%
Subsequent regional recurrence		

No	233	90.7%
Yes	24	9.3%
Subsequent distant metastasis		

No	147	57.2%
Yes	110	42.8%

a International Union Against Cancer.

**Table 2:** Genotypic and allelic frequencies of gene polymorphisms in this study.

Gene	Genotypes	No. (%)	Allele	No. (%)
All patients		257 (100)		514 (100)
ERCC1 codon118	*CC*	142 (55.3)		
	*CT*	100 (38.9)	*C*	384 (74.7)
	*TT*	15 (5.8)	*T*	130 (25.3)
XPD codon751	*AA*	190 (73.9)		
	*AC*	66 (25.7)	*A*	446 (75.5)
	*CC*	1 (0.4)	*C*	80 (24.5)
EGFR codon497	*GG*	52 (20.2)		
	*GA*	120 (46.7)	*G*	224 (43.6)
	*AA*	85 (33.1)	*A*	290 (56.4)

**Table 3:** The relationship between genotype distributions of ERCC1, EGFR, XPD and clinicopathological features in 257 colorectal cancer patients.

Characteristics	Total case No.		ERCC1 CC	ERCC1 TC	ERCC1 TT	EGFR GG	EGFR GA	EGFR AA	XPD AA	XPD AC	XPD CC
			142	100	15	52	120	85	190	66	1
			55.3%	38.9%	5.8%	20.2%	46.7%	33.1%	73.9%	25.7%	0.4%
Gender											

Male	139	54.1%	75	54	10	24	69	46	95	43	1
Female	118	45.9%	67	46	5	28	51	39	95	23	0
*p-*value			0.592			0.390			0.068		
Age (years)											

<60	119	46.3%	64	50	5	26	52	41	98	30	0
≥60	138	53.7%	78	50	10	26	68	44	101	36	1
*p-*value			0.438			0.657			0.636		
Location											

Colon	152	59.1%	86	56	10	35	69	48	118	34	0
Rectum	105	40.9%	56	44	5	17	51	37	72	32	1
*p-*value			0.644			0.403			0.155		
Tumor Size											

<5 cm	156	60.7%	88	59	9	29	79	48	118	37	1
≥5 cm	101	39.3%	54	41	6	23	41	37	72	29	0
*p-*value			0.896			0.288			0.497		
Invasive extent											

T1–T2	49	19.1%	32	14	3	13	24	12	34	15	0
T3–T4	208	80.9%	110	86	12	39	96	73	156	51	1
*p-*value			0.249			0.272			0.613		
Lymph node status											

N0	80	31.1%	45	32	3	17	32	31	60	20	0
N1–N3	177	68.9%	97	68	12	35	88	54	130	46	1
*p-*value			0.630			0.316			0.782		
Stage (UICC) a											

II	60	23.3%	33	24	3	13	26	21	44	16	0
III	197	76.6%	109	76	12	39	94	64	146	50	1
*p-*value			0.942			0.837			0.845		
Histologic differentiation											

Well/moderate	207	80.5%	112	83	12	44	96	67	152	54	1
Poorly	50	19.5%	30	17	3	8	24	18	38	12	0
*p-*value			0.726			0.693			0.841		
Vascular invasion											

Negative	148	57.6%	83	57	8	29	68	51	105	42	1
Positive	109	42.4%	59	43	7	23	52	34	85	24	0
*p*-value			0.919			0.854			0.342		
Perineural invasion											

Negative	204	79.4%	109	84	11	43	97	64	146	57	1
Positive	53	20.6%	33	16	4	9	23	21	44	9	0
*p*-value			0.327			0.504			0.226		
Subsequent regional recurrence											

No	233	90.7%	130	92	11	46	109	78	170	62	1
Yes	24	9.3%	12	8	4	6	11	7	20	4	0
*p*-value			0.059			0.809			0.533		
Subsequent distant metastasis											

No	147	57.2%	81	58	8	30	71	46	115	31	1
Yes	110	42.8%	61	42	7	22	49	39	75	35	0
*p*-value			0.942			0.769			0.109		

a International Union Against Cancer.

**Table 4:** The relationship between clinicopathological features and regional recurrence in 257 colorectal cancer patients.

Characteristics	Total cases No.	%	Regional recurrence *N* = 24	No regional recurrence *N* = 233	*p*-value
Gender					

Male	139	54.1%	13	126	0.993
Female	118	45.9%	11	107	
Age (years)					

<60	119	46.3%	13	106	0.417
≥60	138	53.7%	11	127	
Tumor Size					

<5 cm	156	60.7%	14	142	0.803
≥5 cm	101	39.3%	10	91	
Location					

Colon	152	59.1%	16	136	0.431
Rectum	105	40.9%	8	97	
Invasive extent					

T1–T2	49	19.1%	3	46	0.585
T3–T4	208	80.9%	21	187	
Lymph node status					

N0	80	31.1%	4	76	0.163
N1–N3	177	68.9%	20	157	
Stage (UICC) a					

II	60	23.3%	3	57	0.309
III	197	76.6%	21	176	
Histologic differentiation					

Well/moderate	207	80.5%	16	191	0.071
Poorly	50	19.5%	8	42	
Vascular invasion					

Negative	148	57.6%	14	134	0.938
Positive	109	42.4%	10	99	
Perineural invasion					

Negative	204	79.4%	18	186	0.578
Positive	53	20.6%	6	47	
Subsequent distant metastasis					

No	147	57.2%	14	133	0.906
Yes	110	42.8%	10	100	
Survival					

Alive	203	79%	14	189	0.009
Death	54	21%	10	44	

a International Union Against Cancer.

**Table 5:** The relationship between clinicopathological features and regional recurrence in 257 colorectal cancer patients.

Characteristics	Regional recurrence	No regional recurrence	*p*-Value	OR (CI)	PPV a	NPV b
ERCC1 codon118T/T combined with XPD codon751A/A genotypes	4	8	0.018	5.625 (1.557–20.32)	0.333	0.918
Others	20	225				
ERCC1 codon118T/T combined with EGFR codon497G/G genotypes	2	1	0.024	21.091 (1.838–241.965)	0.667	0.913
Others	22	232				

a PPV: positive predictive value;

b NPV: negative predictive value.

**Table 6:** Characteristics of the studied polymorphisms with primer sequences and restriction enzymes.

Gene	Primer sequence	Restriction enzyme	Polymorphism
ERCC1	[F]:G5′-GCAGAGCTCACCTGAGGAAC-3′	BsrD1	Asn118Asn
	[R]:G5′-GAGGTGCAAGAAGAGGTGGA-3′		
XPD	[F]:G5′-TCTGCAGGAGGATCAGCTG-3′	PstI	Lys751Gln
	[R]:G5′-GCAAGACTCAGGAGTCAC-3′		
EGFR	[F]:G5′-TGCTGTGACCCACTCTGTCT-3′	BsrN1	Arg497Lys
	[R]:G5′-CCAGAAGGTTGCACTTGTCC-3′		
